# Clinical Prognosis in Neonatal Bacterial Meningitis: The Role of Cerebrospinal Fluid Protein

**DOI:** 10.1371/journal.pone.0141620

**Published:** 2015-10-28

**Authors:** Jintong Tan, Juan Kan, Gang Qiu, Dongying Zhao, Fang Ren, Zhongcheng Luo, Yongjun Zhang

**Affiliations:** 1 Xinhua Hospital, Shanghai Jiaotong University School of Medicine, Shanghai, China; 2 Ninth People’s Hospital, Shanghai Jiaotong University School of Medicine, Shanghai, China; 3 Children's Hospital of Shanghai, Shanghai Jiaotong University School of Medicine, Shanghai, China; 4 MOE and Shanghai Key Laboratory of Children’s Environmental Health, Shanghai, China; Midwestern University, UNITED STATES

## Abstract

Neonates are at high risk of meningitis and of resulting neurologic complications. Early recognition of neonates at risk of poor prognosis would be helpful in providing timely management. From January 2008 to June 2014, we enrolled 232 term neonates with bacterial meningitis admitted to 3 neonatology departments in Shanghai, China. The clinical status on the day of discharge from these hospitals or at a postnatal age of 2.5 to 3 months was evaluated using the Glasgow Outcome Scale (GOS). Patients were classified into two outcome groups: good (167 cases, 72.0%, GOS = 5) or poor (65 cases, 28.0%, GOS = 1–4). Neonates with good outcome had less frequent apnea, drowsiness, poor feeding, bulging fontanelle, irritability and more severe jaundice compared to neonates with poor outcome. The good outcome group also had less pneumonia than the poor outcome group. Besides, there were statistically significant differences in hemoglobin, mean platelet volume, platelet distribution width, C-reaction protein, procalcitonin, cerebrospinal fluid (CSF) glucose and CSF protein. Multivariate logistic regression analyses suggested that poor feeding, pneumonia and CSF protein were the predictors of poor outcome. CSF protein content was significantly higher in patients with poor outcome. The best cut-offs for predicting poor outcome were 1,880 mg/L in CSF protein concentration (sensitivity 70.8%, specificity 86.2%). After 2 weeks of treatment, CSF protein remained higher in the poor outcome group. High CSF protein concentration may prognosticate poor outcome in neonates with bacterial meningitis.

## Introduction

Neonates are at high risk of meningitis and of resulting neurologic complications [1]. Despite the availability of newer and more potent antibiotics, the outcome of neonatal bacterial meningitis remains unsatisfactory. Neonatal bacterial meningitis is associated with significant mortality and devastating neurological sequelae, including sensorineural hearing loss, seizures, motor disorders, mental retardation and behavioral problems [2]. Signs of bacterial meningitis are often subtle in the neonates; thus, the diagnosis of meningitis must be made by cerebrospinal fluid (CSF) examination. Currently, a positive CSF culture remains the golden standard for the diagnosis of neonatal bacterial meningitis in clinical practice. However, it may become negative within hours of antibiotic administration. Additionally, CSF culture has been shown to be of poor sensitivity for the diagnosis of bacterial meningitis. Therefore, clinicians must also rely on CSF glucose, white blood cell (WBC) count and protein to determine the presence of meningitis [3,4].

Early recognition of neonates at risk of poor prognosis would be helpful in providing timely management and treatment to improve outcome [5], and to identify individuals who warrant early follow-up and intervention. Several risk factors associated with a poor clinical outcome of bacterial meningitis in children and adults have been identified in previous studies, including the presence of seizures, coma, hypotension, respiratory distress, hypoglycorrhachia, leukopenia or, thrombocytopenia, and delay in the initiation of antibiotic therapy [6–10]. However, there is a lack of data on prognostic factors in bacterial meningitis in neonates.

The aim of the present study was to identify possible risk factors that may predict prognosis in neonates with bacterial meningitis. Moreover, we wanted to clarify whether the CSF parameters are independently associated with poor prognosis.

## Methods

### Patients and data

From January 2008 to June 2014, we enrolled 232 term neonates with bacterial meningitis admitted to 3 neonatology departments in Shanghai, China, including Xinhua Hospital, Ninth People’s Hospital and Children's Hospital of Shanghai. Neonatal bacterial meningitis was defined as clinical symptoms and signs of meningitis plus either a positive culture in the cerebrospinal fluid (CSF), or CSF WBC of >20×10^6^/L in conjunction with a positive blood culture [11,12]. Inclusion criteria were: 1) diagnosis of bacterial meningitis; 2) gestational age (GA) ≥ 37 weeks; 3) age at diagnosis ≤ 28 days. The exclusion criteria were coexisting intrapartum asphyxia, major congenital anomalies, genetic condition or chromosomal abnormality. The study was approved by the Research Ethical Committees of Xinhua Hospital, Ninth People’s Hospital and Children's Hospital of Shanghai. All parents or legal guardians of recruited infants signed an agreement after being disclosed to necessary information on this study, and had given written informed consent.

Outcome data were determined on the day of discharge from hospital or at a postnatal age of 2.5 to 3 months. The clinical status was evaluated with the Glasgow Outcome Scale (GOS) which ranges from 1 to 5: 1 = death; 2 = persistent vegetative state; 3 = severe disability; 4 = moderate disability; 5 = good recovery [13]. Moderate or severe disability was defined as any of the following conditions: spasticity, muscle weakness and immobility in one or more limbs; microcephaly; hydrocephalus; seizure disorder; hearing loss. For the purpose of this analysis, patients with score 5 were defined as having good outcome while patients with score 1 to 4 were defined as having poor outcome.

### Statistical analysis

All statistical analyses were performed using SPSS 16.0 (SPSS Inc., Chicago, IL, USA). Chi square test or Fisher’s exact test was used for categorical variable comparisons. Student's t test or Mann-Whitney test was used to compare continuous variables. Multivariate logistic regression was used to assess the predictors of poor outcome (prognosis) in neonatal meningitis. The cut-off point for CSF protein to predict prognosis in neonatal bacterial meningitis was calculated according to receiver operating characteristic (ROC). Area under the curve (AUC) and sensitive-specific values with 95% confidence intervals were calculated. Results are presented as mean ± standard deviation or n and percentage. *P* values <0.05 were considered statistically significant.

## Results

### Clinical characteristics

Among the 232 patients with meningitis, 88 patients were male and 144 were female, all were born at term. The mean age at onset of meningitis was 13.0 ± 7.7 days. 92 (39.7%) neonates were born by cesarean section (CS), and 140 (60.3%) neonates were vaginal deliveries. The mean birth weight was 3072 ± 422 g. There were 10 neonates with low birth weight (< 2500 g) and 2 neonates with high birth weight (> 4000 g). The infection was observed in 29 (12.5%) infants within the 3 days of life (early onset infection), including 9 (3.9%) on the first day. The average duration of hospital stay was 28.6 ± 14.3 days (range: 1 to 93 days). The overall mortality was 3.0% (7 of 232 patients).

The predisposing factors for neonatal bacterial meningitis included maternal fever greater than 38°C in 38 (16.4%) cases and prolonged rupture of membranes over 12 h in 45 (19.4%) cases. In one case, the cord was cut using a pair of contaminated scissors. 30 (12.9%) cases of vaginal colonization with group B streptococcus were noted. Furthermore, 17 cases had other purulent infections (4 with septic arthritis, 2 with suppurative parotitis, and 11 with omphalitis).

Bacteriological confirmation of meningitis was obtained in 232 cases. Positive cultures of blood and cerebrospinal fluid were obtained in 95 (40.9%) cases and 169 (72.8%) cases, respectively, including 32 (13.7%) cases with a positive culture in both blood and cerebrospinal fluid. Bacteria identified as the causal agent for meningitis were: E. coli, 73 cases; Group B streptococcus, 65 cases; Coagulase negative Staphylococci, 24 cases; Streptococcus pneumoniae, 18 cases; Pseudomonas Aeruginosa, 16 cases; Corynebacterium, 12 cases; Listeria Monocytogenes, 10 cases; Haemophilus influenzae, 8 cases; Meningococcus, 2 cases; Proteusbacillus vulgaris, 2 cases; and Enterococcus feces, 2 case.

### Glasgow Outcome Scale and Neurological Complications

According to the definition of GOS, patients were classified into two outcome groups: good (167 cases, 72.0%, GOS = 5) or poor (65 cases, 28.0%, GOS = 1–4). The poor outcome group included mortality in 7 patients, severe disability in 23 patients and moderate disability in 35 patients.

Neonatal meningitis has been associated with a variety of neurologic complications [14]. In our study, the neurologic complications in poor outcome patients included subdural effusion (15, 23.1%), ependymitis (17, 26.2%) and brain abscess (4, 6.2%). However there were only 8 (4.8%) cases of subdural effusion and none of ependymitis or brain abscess in good outcome patients. It seems that patients with poor outcome had more complications than those with good outcome. (p<0.05)

### Clinical features, Blood and CSF findings

The clinical features of neonates with bacterial meningitis are summarized in Table 1. Overall, fever excluding environmental causes (195, 84.1%) and jaundice with a bilirubin level of more than 85 μmol/L (91, 39.2%) were the two leading presentations, followed by poor feeding (87, 37.5%) and drowsiness (79, 34.1%). The common antecedent illnesses were pneumonia (102, 44.0%), sepsis (95, 40.9%) and diarrhea (30, 12.9%).

**Table 1:** Baseline characteristics in 232 term neonates with bacterial meningitis by prognosis (poor or good outcome*) and predictor for the poor outcome.

		Good outcome*(n = 167)	Poor outcome*(n = 65)	P value	Multivariate logistic regression analysis	
					OR (95% CI)	P value
Sex, male(n)		59(35.3%)	29(44.6%)	0.19		
Birth weight <2500g(n)		7(4.2%)	3(4.6%)	1.00		
Delivery mode, C-Section(n)		59(35.3%)	33(50.8%)	0.03	1.84 (0.67–5.08)	0.24
Age at onset(days)		13.5(7.8)	11.7(7.3)	0.10		
Hospital stay(days)		28.0(12.4)	30.3(18.2)	0.07		
Clinical symptoms and signs	Fever(n)	139(83.2%)	56(86.2%)	0.59		
	Apnea (n)	4(2.4%)	12(18.5%)	<0.001	3.12(0.33–29.72)	0.32
	Drowsiness(n)	40(24.0%)	39(60.0%)	<0.001	1.35(0.46–4.00)	0.58
	Poor feeding(n)	40(24.0%)	47(72.3%)	<0.001	3.83(1.22–12.05)	0.02
	Vomiting(n)	7(4.2%)	7(10.8%)	0.07		
	Jaundice(n)	73(43.7%)	18(27.7%)	0.03	0.40(0.13–1.23)	0.11
	Seizures (n)	24(14.4%)	13(20.0%)	0.29		
	Bulging fontanelle(n)	5(3%)	8(12.3%)	0.01	1.81(0.26–12.65)	0.55
	Irritability(n)	10(6%)	18(27.7%)	<0.001	1.22(0.31–4.77)	0.78
	Hepatosplenomegaly(n)	8(4.8%)	4(6.2%)	0.74		
Antecedent illness	Sepsis(n)	74(44.3%)	21(32.3%)	0.10		
	Pneumonia(n)	61(36.5%)	41(63.1%)	<0.001	3.37(1.15–9.84)	0.03
	Impetigo neonatorum(n)	2(1.2%)	3(4.6%)	0.14		
	Diarrhea(n)	28(16.8%)	2(3.1%)	0.005	0.31(0.04–2.45)	0.27
Blood parameters	White blood cell(×10^9^/L)	16.7(6.5)	15.3(6.5)	0.15		
	Hemoglobin(g/L)	142.0(29.5)	125.3(29.2)	<0.001	0.62(0.37–1.04)	0.07
	Platelet(×10^9^/L)	298.3(113.2)	274.9(159.0)	0.28		
	Thrombocytocrit (%)	0.47(0.19)	0.55(0.49)	0.11		
	Mean platelet volume(fl)	10.5(1.3)	11.5(1.5)	<0.001	1.62(0.92–2.84)	0.10
	Platelet distribution width(fl)	12.5(2.1)	13.4(2.2)	0.004	1.08(0.63–1.86)	0.77
	C-reaction protein >8mg/L(n)	111(66.5)	56(83.6)	<0.001	1.45(0.53–3.95)	0.47
	Procalcitonin≥0.05ug/L(n)	117(70.1%)	62(95.4%)	<0.001	2.37(0.64–8.78)	0.20
CSF parameters	WBCs (×10^6^/L)					
	Mean ± SD	212.8(351.2)	886.0(2700.4)	0.15		
	<100	96(57.5%)	31(47.7%)	0.39		
	100∼1000	60(35.9%)	28(43.1%)			
	>1000	11(6.6%)	6(9.2%)			
	CSF polykaryocyte (%)	55.6(20.5)	52.2(20.0)	0.09		
	CSF glucose(mmol/L)	2.3(1.1)	1.9(1.5)	<0.001	0.90(0.61–1.33)	0.58
	CSF protein(mg/L)	1387.0(781.6)	2549.2(1019.7)	<0.001	4.07(2.33–7.11)	<0.001

Data are n (%) or mean (SD)

CSF = cerebrospinal fluid; WBC = white blood cell.

*The prognosis outcome was defined by clinical status on hospital discharge or at a postnatal age about 2.5 to 3 months. Using Glasgow Outcome Scale (GOS): 1 death, 2 persistent vegetative state, 3 severe disability, 4 moderate disability, 5 good recovery; GOS = 1–4: poor outcome, GOS = 5: good outcome.

Peripheral blood and CSF cyto-biochemical findings are also summarized in Table 1. WBC varied between 2.45 and 35.98 ×10^9^/L with a mean of 16.28×10^9^/L, including 67 cases with a leukocytosis more than 20.0×10^9^/L and 2 cases with a leucopenia less than 4.0×10^9^/L. Anemia with a hemoglobin less than 145 g/L in 151 cases. 72.0% of cases had a C-reaction protein (CRP) level over 8 mg/dl. Procalcitonin (PCT) was 0.05 μg / L or over in 77.2% of cases.

CSF cell count varied between 11 and 17870×10^6^/L with a median of 80×10^6^/L, including 105 cases with the number of cells more than 100×10^6^/L. CSF polykaryocyte (%) > 50% was observed in 65.9% of cases. Protein concentration varied between 438 and 4750 mg/L, with a median of 1400 mg/L. CSF glucose <1.5 mmol/L in 25.0% of cases, and CSF glucose / blood glucose ratio <0.5 in 62.1% of cases.

Comparison between the two groups showed there was no statistically significant difference in the sex, age at onset, birth weight, and hospital stay between the two groups. However, neonates with good outcome had less frequent apnea, drowsiness, poor feeding, bulging fontanelle, irritability and more severe jaundice compared to neonates with poor outcome (*p*<0.05). The good outcome group also had less pneumonia and more diarrhea than the poor outcome. Besides, there were statistically significant differences in hemoglobin, mean platelet volume, platelet distribution width, C-reaction protein, procalcitonin, CSF glucose and CSF protein. (Table 1)

### Predictor factors of poor outcome in neonatal bacterial meningitis

Multivariate logistic regression analyses were performed including variables found by univariate analyses to be associated with poor outcome with *p* <0.05. Poor feeding (OR of 3.83, 95% CI = 1.22–12.05, *p* = 0.02), pneumonia (OR of 3.37, 95% CI = 1.15–9.84, *p* = 0.03) and CSF protein (OR of 4.07, 95% CI = 2.33–7.11, *p*<0.001) were the predictors of poor outcome. (Table 1)

**Fig 1:**
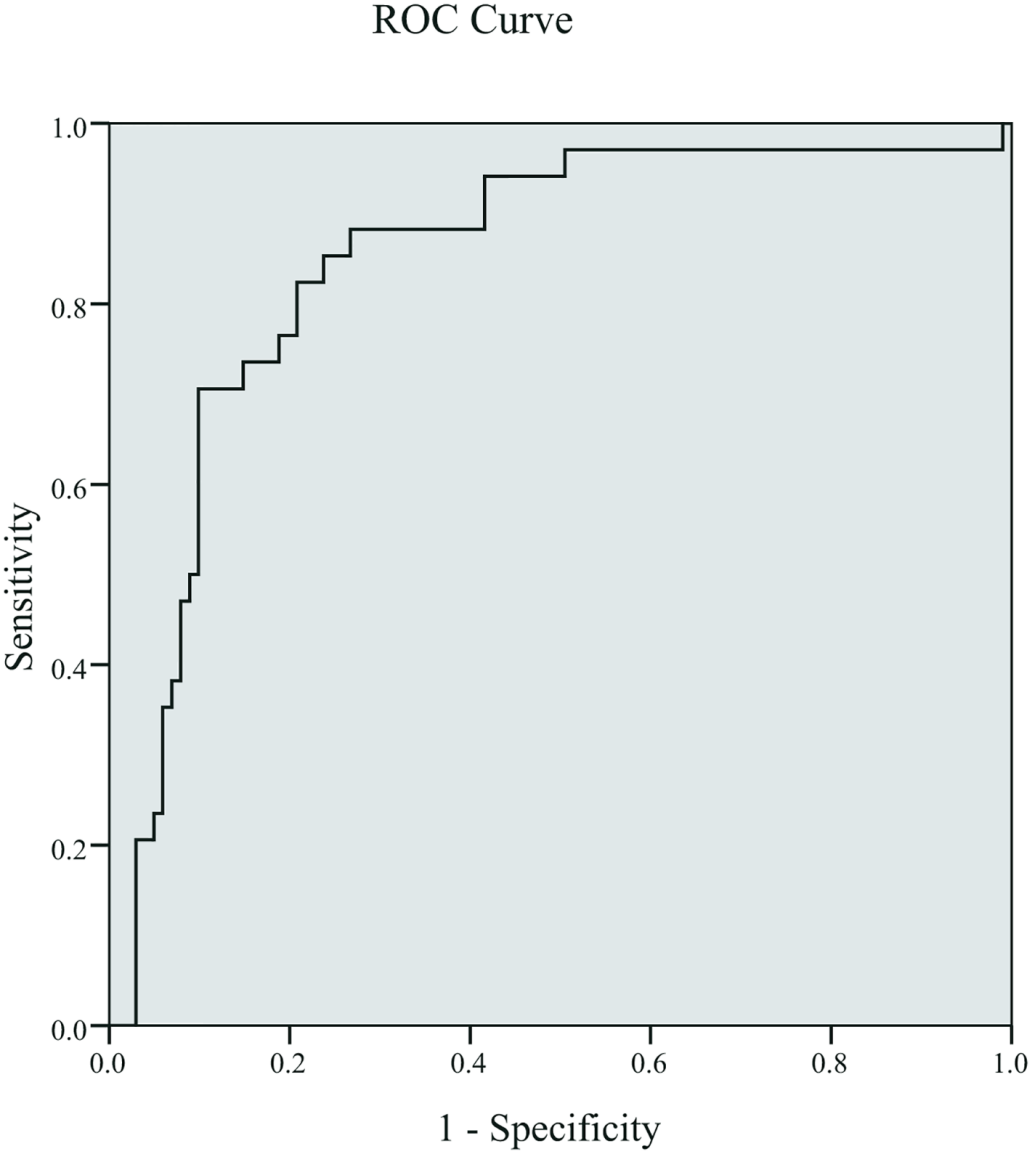
Receiver operating characteristic curves for CSF protein in predicting outcomes (poor, good) in neonates with bacterial meningitis. Sensitivity (true positive rate) is plotted against 1-specificity (false positive rate). The area under the curve was 0.842 for the best cutoff value at 1880 (specificity = 70.8%, sensitivity = 86.2%).

We next conducted ROC curve analysis to predict severity of bacterial meningitis by CSF protein. The AUC predicting poor outcome was 0.842. The best cut-off for predicting poor outcome was 1880 mg/L for CSF protein (sensitivity 70.8%, specificity 86.2%). (Fig 1)

### Changes in CSF parameters after treatment

Despite the availability of routine effective treatment, unfortunately, 7 patients died due to the infection within seven days of admission. The remaining patients had at least 2 weeks of antibiotic treatment. We compared the CSF parameters before and after 2 weeks of treatments. CSF protein content remained higher and CSF glucose remained lower in the poor vs. the good outcome groups (*p*<0.001). (Table 2)

**Table 2:** CSF parameters in 225 term neonates with bacterial meningitis after two weeks of antibiotic treatment*.

Variable	Good outcome (n = 167)	Poor outcome*(n = 58)	P value
WBCs (×10^6^/L)	14.2(19.8)	20.1(27.0)	0.90
CSF glucose(mmol/L)	3.1(0.6)	2.7(0.6)	<0.001
CSF protein(mg/L)	922.7(428.3)	1582.5(686.8)	<0.001

CSF = cerebrospinal fluid; WBC = white blood cell.

*The poor outcome had excluded 7 patients with Glasgow Outcome Scale (GOS) = 1

## Discussion

Newborns are especially vulnerable to infection. The cellular and humoral immune systems are immature, including the phagocytic function. The incidence of meningitis ranges from 0.2 to 6.1 cases per 1,000 live newborns [15,16]. The clinically signs and symptoms of neonatal meningitis are largely subtle. In our study, fever (84.1%), jaundice (39.2%), poor feeding (37.5%) and drowsiness (34.1%) were the common symptoms as in previous studies of neonatal meningitis [17,18]. However, these features could not help to establish an early diagnosis of meningitis.

The incidence of meningitis confirmed by culture in the CSF was not high. But there is a strong association of meningitis and sepsis with positive blood cultures [19]. Therefore, many studies defined meningitis by a positive culture from the blood and abnormal cerebrospinal fluid parameters [2,6,20]. We used these definitions for patients' inclusion criteria in our study as well. Moreover, coliforms and Group B streptococci are the common causal bacteria in meningitis in neonates which is confirmed in our study [21,22].

Early recognition of meningitis infants at risk of poor prognosis would be helpful in providing prompt management and identifying those who warrant long-term follow-up and early intervention [23–25]. Previous studies showed a number of possible early markers of poor prognosis in neonatal meningitis. For example, Grimwood found that symptoms>24 h, seizures after 72 h in hospital and focal neurological signs are independent risk factors of bacterial meningitis [20]. Two retrospective studies showed that seizures, thrombocytopenia, high CSF protein, and low CSF glucose concentration were important prognostic factors of complications in neonatal meningitis [5,26]. We found the poor outcome group had more neurological symptoms and sequelae than the good outcome group including bulging fontanelle, irritability, subdural effusion, ependymitis and brain abscess.

Besides, we found that the poor feeding, pneumonia and CSF protein were predictive of poor outcome in our study. Weber et al found that reduced feeding ability may be an independent predictor of severe disease [27]. However, it had limited specificity in distinguishing from other severe diseases such as sepsis, meningitis, hypoxemia, or radiologically proven pneumonia. Ben et al found that respiratory distress was one of the main factors of a poor prognosis in neonatal bacterial meningitis [28]. We found that pneumonia was related to poor outcome in neonatal meningitis.

It should be emphasized that CSF protein in the poor outcome group was extremely high in our study. The level of cerebrospinal fluid protein content significantly influenced patient prognosis. Though previous study showed that high CSF protein was associated with poor prognosis in childhood bacterial meningitis [29], no studies have shown the exactly level of CSF protein is predictive of poor outcome in neonatal meningitis. However, we found that CSF protein ranged 438–4750 mg/L (median of 1400 mg/L), similar to Garges HP`s study reporting that CSF protein in neonates with bacterial meningitis ranged 410–19640 mg/L (median: 2730 mg/L) [4]. Furthermore, we found that the best cutoff value was 1880 mg/L (specificity = 70.8%, and specificity = 86.2%) for predicting poor prognosis in neonatal meningitis.

It has been recognized that WBCs, immunoglobulins, and complements are normally sparse or absent in CSF. At the initial stage of neonatal meningitis, micro-organisms can cross the blood—brain barrier via microbial interactions with host receptors, and replicate within the subarachnoid space concomitantly. Later bacteria release endotoxins, teichoic acid, and other substances that trigger an inflammatory response with mediators such as WBCs and TNF resulting in increasing protein levels in CSF. Besides, the production of cytokines leads to the attraction and activation of polymorphonuclear leukocytes and the production of high amounts of reactive oxygen species. These free radicals are highly reactive and may cause impairment of lipids, proteins, carbohydrates or nucleic acids, thus increasing the risk of poor sequelae. Because of the high lipid content in the brain and low cerebral antioxidant defense, the central nervous system is particularly susceptible to the deleterious properties of oxidative stress [30,31]. So we speculated that the high protein in CSF was related to the intensity of the inflammatory response. The inflammatory cascade in meningitis may result in deleterious neurological morbidity. Furthermore, our study demonstrated that after 2 weeks of therapy, the poor outcome group still had higher CSF protein levels, indicating that high CSF protein content may be of value in selecting patients for more intensive therapy and in identifying candidates for new treatment strategies.

In summary, CSF protein may be a biomarker of severe CNS infection with poor prognosis for identifying patients for timely and intensive treatment.
